# 3D nanoprinting of PDMS microvessels with tailored tortuosity and microporosity *via* direct laser writing†

**DOI:** 10.1039/d4lc01051e

**Published:** 2025-03-12

**Authors:** Xin Xu, Yunxiu Qiu, Chen-Yu Chen, Molly Carton, Paige M. R. Campbell, A. Muhaymin Chowdhury, Bidhan C. Bandyopadhyay, William E. Bentley, Bryan Ronain Smith, Ryan D. Sochol

**Affiliations:** a Department of Mechanical Engineering, University of Maryland College Park MD 20742 USA rsochol@umd.edu; b Institute for Quantitative Health Science and Engineering, Department of Chemical Engineering and Material Science, Michigan State University East Lan-sing MI 48824 USA; c Fischell Department of Bioengineering, University of Maryland College Park MD 20742 USA; d Robert E. Fischell Institute for Biomedical Devices, University of Maryland College Park MD 20742 USA; e Veterans Affairs Medical Center Washington, D.C. 20422 USA; f Department of Biomedical Engineering, Michigan State University East Lansing MI 48824 USA; g Maryland Robotics Center, University of Maryland College Park MD 20742 USA; h Institute for Systems Research, University of Maryland College Park MD 20742 USA

## Abstract

Microvessels (*e.g.*, capillaries) are ubiquitous throughout human anatomy, yet recreating their three-dimensional (3D) microfluidic and architectural sophistication at biologically accurate length scales has remained a critical challenge. To overcome this barrier, here we report a hybrid additive manufacturing—or “3D printing”—strategy in which “Two-Photon Direct Laser Writing (DLW)” is used to nanoprint microvessels of arbitrary design directly atop “Liquid-Crystal Display (LCD)” 3D-printed microfluidic chips. Fabrication results indicated effective production of 100 μm-diameter 3D polydimethylsiloxane (PDMS) microfluidic vessels with 5 μm-thick walls—featuring arrays of pre-designed 5 μm-diameter micropores—as well as three discrete spiralled, intertwined microvessels. Experimental results with MDA-MB-231 epithelial breast cancer cells revealed the ability for the 3D PDMS microvessels to support cell culture. In combination, these results suggest that the presented strategy for 3D nanoprinting PDMS microvessels with custom-designed architectures and microporosity offers a promising pathway to enable new classes of “organ-on-a-chip (OOC)” systems for wide-ranging biomedical applications.

## Abstract 2

A novel two-photon direct laser writing-based hybrid strategy for 3D nanoprinting microfluidic vessels with sophisticated 3D architectures and custom-designed micropores.

## Graphical abstract

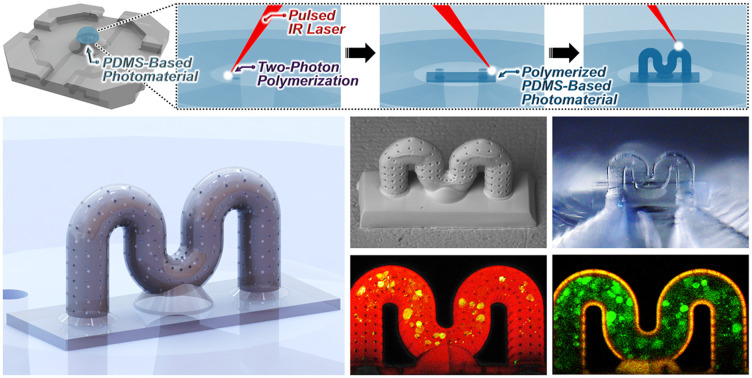

## Introduction

Microvessels represent vital building blocks across all organ systems of the human body.^1–3^ The ability to engineer biologically relevant microvessels *in vitro* could have profound implications for a diversity of biomedical applications, including drug screening, disease modelling, and personalized medicine.^4–6^ In many respects, however, the critical barrier impeding such advancements is fundamentally a manufacturing one: conventional microfluidics manufacturing techniques are poorly suited for replicating the physical complexity inherent to microvessels *in vivo*.^7–10^ Ideally, methods for fabricating microvessels *in vitro* should be able to reproduce four key characteristics of their *in vivo* counterparts: (i) local microcurvature (*e.g.*, fully circular vessel cross sections of ≤100 μm in diameter), (ii) global tortuosity (*e.g.*, 3D winding or curvature of the overall vessel path), (iii) mechanically soft vessel material, and (iv) thin (*e.g.*, ≤10 μm) vessel walls with tailored microporosity (*e.g.*, ≤5 μm pores).^11–13^ Although microfluidic fabrication strategies reported previously have not yet facilitated all of these biophysical characteristics simultaneously, researchers have developed and demonstrated a wide range of approaches for manufacturing microfluidic vessel-mimetic structures as a means to realize microphysiological—or “organ-on-a-chip (OOC)”—systems designed to emulate tissue- and organ-level processes *in vitro*.^14–17^

The majority of current protocols for producing microfluidic systems comprising microvessel-like components fall into four principal categories. The most prominent technique extends multi-layer soft lithography to achieve polydimethylsiloxane (PDMS) devices with two parallel microchannels separated by a thin, flexible, and microporous membrane, but with the caveat that the channels are planar (*i.e.*, flat) and include rectangular cross sections and flow profiles.^18–24^ To fabricate devices with circular microchannel cross sections, a second approach involves casting cylindrical templates (*e.g.*, rods, pins, fishing lines or wires) such that when the templates are removed, there remain straight microchannels with circular cross-sectional profiles surrounded by extracellular matrix (ECM).^25–31^ The third main approach is similar to the second, but with the key difference that 3D-printed sacrificial inks—deposited by the material extrusion technique, “Direct Ink Writing (DIW)”—serve as the template being cast (and then evacuated).^32–35^ Although such methods can enable considerable vessel tortuosity, the reliance on nozzle-based printing leads to difficulties in resolving microvessels with fully circular (rather than semi-circular) cross-sectional profiles.^32–35^ Lastly, investigators have reported vessel self-assembly techniques by which living cells, such as endothelial cells, form tubular networks of microvessels *in vitro* akin to vasculogenesis and angiogenesis *in vivo*; however, such methods are not only time-intensive (*e.g.*, one week for vessel assembly), but also inherently lead to high variability in assembled vessel networks from device to device.^36–42^ Consequently, new strategies for manufacturing microvessels that satisfy all of the aforementioned biophysical and bioarchitectural criteria in a controlled and repeatable manner are in critical demand.

Among the numerous additive manufacturing technologies, “Two-Photon Direct Laser Writing (DLW)” offers unparalleled geometric versatility and print speeds at length scales down to the 100 nm range.^43–45^ DLW entails scanning a tightly focused femtosecond laser within a photocurable material to initiate two-photon polymerization (2PP) in a point-by-point and/or layer-by-layer process to, ultimately, produce 3D micro- or nanostructured parts comprising cured photomaterial.^46–48^ For DLW-based microfluidic systems, however, the ability to deliver and/or retrieve fluids necessitates macro-to-micro interfaces (*e.g.*, inlet and outlet ports), which can be exceedingly time- and cost-intensive to 3D print *via* DLW.^49–51^ To overcome this drawback for microvessel fabrication, we previously reported an “*in situ* DLW (*is*DLW)” approach—*i.e.*, protocols in which microfluidic structures are printed directly inside of a fully enclosed microfluidic channels—for 3D printing fully intertwined microvessels that comprised <10 μm inner diameters (IDs) and 2 μm-thick walls.^52^ Unfortunately, because *is*DLW protocols are performed inside of enclosed microdevices, the laser must pass through the device substrate (*e.g.*, a thin thermoplastic sheet or a glass slide) to reach the photomaterial inside the microchannel, which restricts the total print height to approximately 100 μm.^52^ Consequently, *is*DLW approaches are ill suited for microvessel fabrication in many cases. Recently, researchers have reported alternative “*ex situ* DLW (*es*DLW)” techniques that instead entail DLW-printing 3D microfluidic structures directly atop meso/macroscale fluidic components (*e.g.*, glass capillaries and fluidic tubing), with applications ranging from microneedle array-based drug delivery to soft microrobotics.^53–59^ Because the microstructures are printed in unenclosed scenarios, the height constraints of *is*DLW can be circumvented; however, the potential of *es*DLW for microvessel fabrication has not yet been explored. Thus, here we investigate the utility of *es*DLW as a means for enabling PDMS microvessels to be manufactured with customized 3D architectures and microporosity.

## Experimental

### Concept

**Fig. 1:**
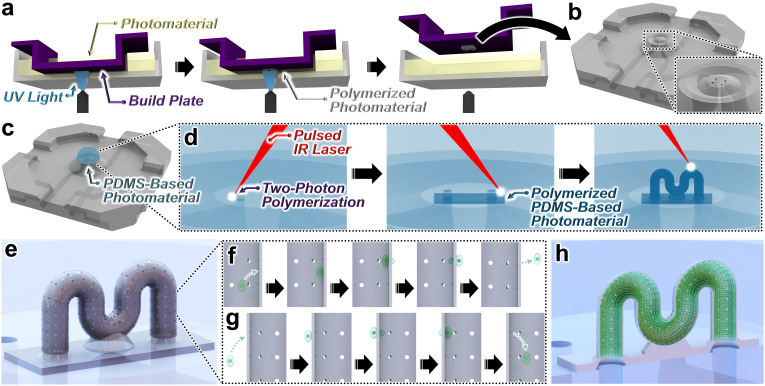
Conceptual illustrations of the hybrid additive micro-nanomanufacturing strategy for three-dimensional (3D) polydimethylsiloxane (PDMS)-based microvessel fabrication. (a) “Vat Photopolymerization (VPP)” of the base microfluidic chip, *e.g.*, *via* “Liquid Crystal Display (LCD)” 3D printing. (b) The base microfluidic chip with externally accessible top microfluidic ports. (c) PDMS-based photomaterial deposited onto the top microfluidic ports of the base microfluidic chip. (d) “Two-Photon Direct Laser Writing (DLW)” of a demonstrative 3D microfluidic vessel structure with pre-designed micropores printed directly atop—and fully fluidically interfaced with—the base microfluidic chip. A tightly focused pulsed infrared (IR) laser is scanned within the PDMS-based photomaterial to initiate two-photon polymerization (2PP) point by point, layer by layer, in designated locations. (e) The developed microvessel (atop the base microfluidic chip) comprising cured (crosslinked) PDMS-based photomaterial. (f–h) Examples of potential microvessel use cases, such as biomedical research studies and applications based on: (f) extravasation, (g) intravasation, and (h) vessel cellularisation (cross-sectional views).

The presented hybrid additive micro-nanomanufacturing strategy for microvessel fabrication involves two stages (Fig. 1). First, a “Vat Photopolymerization (VPP)” technique—in this work, “Liquid Crystal Display (LCD)” 3D printing—is used to additively manufacture a base microfluidic chip (Fig. 1a and b). LCD 3D printing entails spatially controlled layer-by-layer crosslinking of a photocurable material *via* an array of light-emitting diodes (LEDs) that project ultraviolet (UV) light through an LCD screen into a vat of liquid-phase photomaterial (Fig. 1a).^60,61^ Initially, the base microfluidic chip is fabricated while adhered to the build plate (Fig. 1a); however, following completion of the 3D printing process, the microchip can be removed from the build plate for development and subsequent use (Fig. 1b). The base microfluidic chip serves as an intermediary component for macro-to-micro fluidic interfacing by including externally accessible top microfluidic ports (100 μm in diameter) that each connect *via* a distinct internal 3D microchannel to a corresponding lateral port designed for a 21-gauge (1.0 mm in diameter) catheter coupler that can be manipulated by hand (Fig. 1b).

In the second stage, a liquid-phase PDMS-based photomaterial—IP-PDMS (Nanoscribe GmbH & Co. KG, Germany)—is placed onto the externally accessible top microfluidic ports of the base microfluidic chip (Fig. 1c) and then microvessels of interest are 3D printed directly atop the ports *via es*DLW (Fig. 1d). Due to the geometric versatility and feature resolutions of DLW, the microvessels can be constructed with arbitrary 3D designs, thereby affording high control not only over the microvessel cross-sectional profile and tortuosity, but also in customizing the microvessel walls with pre-designed micropores (Fig. 1e). Accordingly, the resulting 3D PDMS microvessels can be used for diverse biomedical research and applications ranging from extravasation (Fig. 1f) and intravasation (Fig. 1g) studies to those based on cellularized vessels (Fig. 1h).

### Base microfluidic chip fabrication *via* “Liquid Crystal Display (LCD)” 3D printing

To fabricate the base microfluidic chips, first, the 3D microchips were modelled using the computer-aided design (CAD) software, SolidWorks (Dassault Systèmes, France). The CAD models were exported as STL files and then imported into the slicing software, CHITUBOX (China), for compatibility with the ELEGOO Mars 3 LCD 3D printer (ELEGOO, China) (ESI† Table S1). The Mars 3 LCD 3D printer (ELEGOO, China) was used for microchip fabrication with the photomaterial, Clear Microfluidic Resin v7.0a (CADworks, Canada). The prints were developed by rinsing with ethanol to remove residual resin and dried with N_2_ gas. Lastly, the prints underwent an additional ultraviolet (UV) curing step for 30 seconds to ensure complete polymerization.

### Microvessel fabrication *via* “*Ex Situ* Direct Laser Writing (*es*DLW)”

All microvessel designs were modelled in SolidWorks CAD software (Dassault Systèmes, France) and then exported in the STL file format. For DLW-based printing with the Nanoscribe Photonic Professional GT2, the STL files were imported into the computer-aided manufacturing (CAM) software, DeScribe (Nanoscribe), for laser writing path generation. Prior to the printing process, PDMS-based photoresist, IP-PDMS (Nanoscribe), was dispensed onto the top ports of the microfluidic chip. The assembly was then loaded into the Nanoscribe Photonic Professional GT2 system for DLW printing using the Dip-in Laser Lithography (DiLL) mode and the 10× objective lens. The *es*DLW process for the single tortuous microvessel was initiated with a 15 μm overlap into the microchip's surface to enhance fluidic sealing, while the DLW print parameters for hatching distance and layer height were both set at 300 nm (ESI† Table S2).

Following the *es*DLW printing process, the full assembly—comprising the 3D microvessel atop the base microfluidic chip—was removed from the printer and immersed in 50 °C isopropyl alcohol (IPA) for 30 minutes and then fresh room-temperature IPA for another 30 minutes to remove any residual (*i.e.*, uncured) photomaterial. Lastly, after the system was allowed to dry under ambient conditions for approximately 3–5 minutes, the device assembly was exposed to UV light for 60 seconds.

For *es*DLW-based printing with the UpNano NanoOne 1000 3D Microfabrication Systems (UpNano GmbH, Vienna, Austria), the STL files were imported into the CAM software, Think3D (UpNano), for laser writing path generation. Prior to the printing process, photoresist—IP-PDMS (Nanoscribe) for the serpentine microvessel and (non-PDMS) UpFlow (UpNano) for the intertwined microvessels—was dispensed onto the top ports of the microfluidic chip. The assembly was then loaded into the UpNano system for DLW-based 3D printing using the Vat mode with 20× and 10× objective lenses for the serpentine microvessel and the intertwined microvessels, respectively. The *es*DLW process was initiated with approximately 10–15 μm overlap into the chip's surface to enhance fluidic sealing. The DLW print parameters for the three intertwined microvessels are presented in ESI† Tables S3 and S4; the detailed settings for the serpentine microvessels are presented in ESI† Tables S5 and S6. Following the *es*DLW printing process, the microvessel printed with IP-PDMS was developed in the same fashion as previously discussed; while the full assembly printed with UpFlow was removed from the printer and immersed in 50 °C isopropyl alcohol (IPA) for 15 minutes and then fresh room-temperature IPA for another 5 minutes to remove any residual photomaterial. Lastly, the system was allowed to dry under ambient conditions (*e.g.*, <5 minutes).

### Optical characterizations

Micrographs captured during the *es*DLW printing process using the Nanoscribe DLW printer were captured using the built-in Carl Zeiss Axio Observer inverted microscope (Zeiss, Germany). Scanning electron microscopy (SEM) images were obtained using a TM4000 Tabletop SEM (Hitachi, Tokyo, Japan). Brightfield and fluorescence micrographs of experimental results were performed using: (i) a Macro Zoom Fluorescence Microscope System (MVX10, Olympus) equipped with X-Cite Illuminators for fluorescence excitation and a DP74 charge-coupled device (CCD) camera (Olympus), and (ii) an Axio Observer.Z1 (Zeiss) inverted microscope connected to a CCD camera (Axiocam 503 Mono, Zeiss).

### Microfluidic experimentation

All microfluidic experiments were conducted using the Fluigent Microfluidic Control System (MFCS) and Flow Rate Platform, along with OxyGen software (Fluigent, France). The side ports of the base microfluidic device were connected to the Fluigent system *via* stainless-steel catheter couplers (21 ga., Instech, Plymouth Meeting, PA) interfaced with fluorinated ethylene propylene (FEP) fluidic tubing (Cole-Parmer, Vernon Hills, IL). For the linear burst-pressure studies, a suspension of fluorescently labelled microbeads (1.0 μm Fluoro-Max Dyed Green Aqueous Fluorescent Particles, Thermo Fisher Scientific, Waltham, MA) was infused into the microvessels as the input pressure (*P*_in_) was increased by 1 kPa per 0.1 seconds from 0 to 100 kPa—*i.e.*, the maximum pressure range for Fluigent LU-FEZ-1000 unit—while the system was monitored under fluorescence microscopy using a Macro Zoom Fluorescence Microscope System (MVX10, Olympus).

For the microfluidic extravasation studies, we infused both a 10% fluorescein-5-isothiocyanate (FITC) solution (Thermo Fisher Scientific) as well as a suspension of fluorescently labelled microbeads (1.0 μm Fluoro-Max Dyed Green Aqueous Fluorescent Particles, Thermo Fisher Scientific) into the microvessels with *P*_in_ set to 0, 5, 10, 15, and 20 kPa while the outlet pressure (*P*_out_) was set to 0, −5, and −10 kPa. We calculated the “Relative Fluorescence Intensity (RFI)” for select “Regions of Interest (ROIs)” corresponding to each pressure set (*i.e.*, *P*_in_ and *P*_out_ combination) as:1
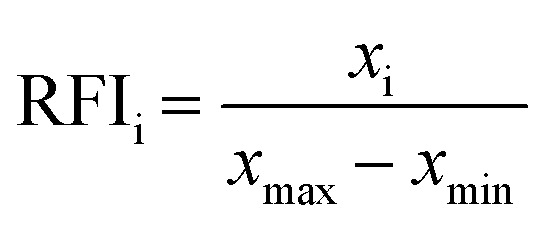
where *x*_i_ is the fluorescence intensity of a single ROI from a single frame of video (ESI† Movie S1), *x*_max_ and *x*_min_ are the maximum and minimum recorded fluorescence intensities, respectively, for all ROIs corresponding to a specific applied vacuum pressure condition (*i.e.*, *P*_out_ = 0, −5, or −10 kPa) (*n* = 90 frames per ROI per distinct set of pressure conditions). This calculation of RFI values normalized the fluorescence results such that the RFI magnitudes ranged from 0% to 100% for each of the three outlet vacuum pressure settings (*i.e.*, *P*_out_ = 0, −5, and −10 kPa).

### Cell experimentation

First, the device was prepared for cellular testing by immersing it in ethanol for 12 hours, followed by immersion in DI water for an additional 12 hours, and then rinsed with fresh DI water for 1 minute. The device was subjected to oxygen plasma treatment at 35 W for 60 seconds with a flow rate of 40 sccm using a Tergeo Plasma Cleaner (PIE Scientific, USA). A 0.2% gelatin solution (porcine skin, Sigma-Aldrich) was infused into the microvessels *via* the side ports of the bulk microchip and incubated at 37 °C overnight. The system was then rinsed with phosphate buffered saline (PBS, Thermo Fisher Scientific) and Dulbecco's modified Eagle's medium (DMEM, Thermo Fisher Scientific). A suspension of MDA-MB-231 cells at a concentration of 1 × 10^7^ cells per mL in culture medium RPMI 1640 (Gibco™) with 10% fetal bovine serum (FBS, Gibco™) and 1% penicillin strep (Gibco™) was loaded into the microvessel *via* the side port of the base microfluidic chip. The microvessel was then soaked in the cell culture medium and cultured at 37 °C in a 5% CO_2_ incubator for cell adhesion and growth. Cell viability was assessed 24 hours after cell seeding with gentle flushing and a 2.5 μg ml^−1^ propidium iodide (PI) staining. The MDA-MB-231 cells are GFP-expressing cells (excitation 475 nm, emission 509 nm) and are excited at 900 nm, while microchannel autofluorescence and PI fluorescence (excitation 532 nm, emission 630 nm) are excited at 1040 nm. A Leica TCS SP8X two-photon microscope (Leica Camera AG, Wetzlar, Germany) was used to captured fluorescence images of the cells. The 3D reconstruction images of the microvessels were generated using LAS X 3D Viewer (Leica).

## Results and discussion

### Microvessel fabrication

**Fig. 2:**
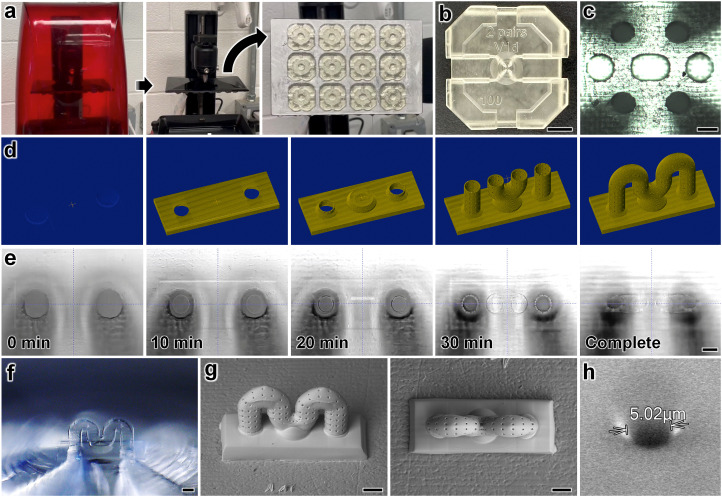
Fabrication results for “*ex situ* DLW (*es*DLW)” 3D printing of a serpentine microvessel with pre-designed micropores atop an LCD 3D-printed base microfluidic chip. (a) Removal of the build substrate following LCD 3D printing of 12 base microfluidic chips simultaneously (see also ESI† Movie S2). (b) Top view of a single base microfluidic chip. Scale bar = 5 mm. (c) Brightfield micrograph of the four externally accessible top fluidic ports of the base microfluidic chip. Scale bar = 100 μm. (d and e) The *es*DLW 3D printing process for fabricating a PDMS microvessel directly atop two of the externally accessible top fluidic ports of the base microfluidic chip. (d) Computer-aided manufacturing (CAM) simulations. (e) Corresponding micrographs captured during the *es*DLW 3D printing process. Total print time ≈ 38 min; scale bar = 100 μm (see also ESI† Movie S3). (f–h) Micrographs of fabricated PDMS microvessels imaged under (f) brightfield microscopy, and (g and h) scanning electron microscopy (SEM). (f and g) Views of complete microvessel structures. Scale bars = 100 μm. (h) Magnified SEM micrograph of a single micropore.

The LCD 3D printing process was completed in less than 30 minutes, with the capacity to produce up to 12 microchips per batch (Fig. 2a; ESI† Movie S2). Fabrication results revealed high fidelity and yield of the base microfluidic chip (Fig. 2b), including the ability to resolve externally accessible top microfluidic ports of approximately 100 μm in diameter (Fig. 2c). It is important to note, however, that a variety of VPP 3D printers—*e.g.*, those based on stereolithography or “Digital Light Processing (DLP)”—could be substituted for fabrication of the base microchip, under the condition that they can resolve sufficiently small microchannel diameters (*e.g.*, ≤100 μm).

To provide an exemplar with which to investigate the presented microvessel fabrication strategy, we designed 3D serpentine microfluidic vessel structures with IDs of 100 μm and wall thicknesses of 5 μm as well as 5 μm-in-diameter micropores arrayed throughout the vessel walls. CAM simulations and corresponding micrographs captured during the *es*DLW 3D printing process are presented in Fig. 2d and e, respectively, with a total print time of less than 38 minutes (ESI† Movie S3). Brightfield (Fig. 2f) and SEM (Fig. 2g) micrographs of fabricated microvessels revealed effective production of the PDMS microvessels, including the arrayed 5 μm-in-diameter micropores (Fig. 2h). We designed these wall thicknesses and micropore sizes with respect to the voxel size associated with the objective lens of the DLW 3D printer (ESI† Table S7); however, these feature sizes could be adjusted by printing with a different objective lens. For example, higher-magnification objective lenses than those used in the present study (*e.g.*, 63×) could enable wall thicknesses as small as 550 nm and micropores on the order of 0.5–1 μm. In addition, we designed the overall footprint of the serpentine microvessel to prevent the need for stitching based on the DLW 3D printer's build size (*i.e.*, field of view, ESI† Table S7). To support applications that benefit from longer microvessels, stitching-based approaches could be investigated in future studies.

**Fig. 3:**
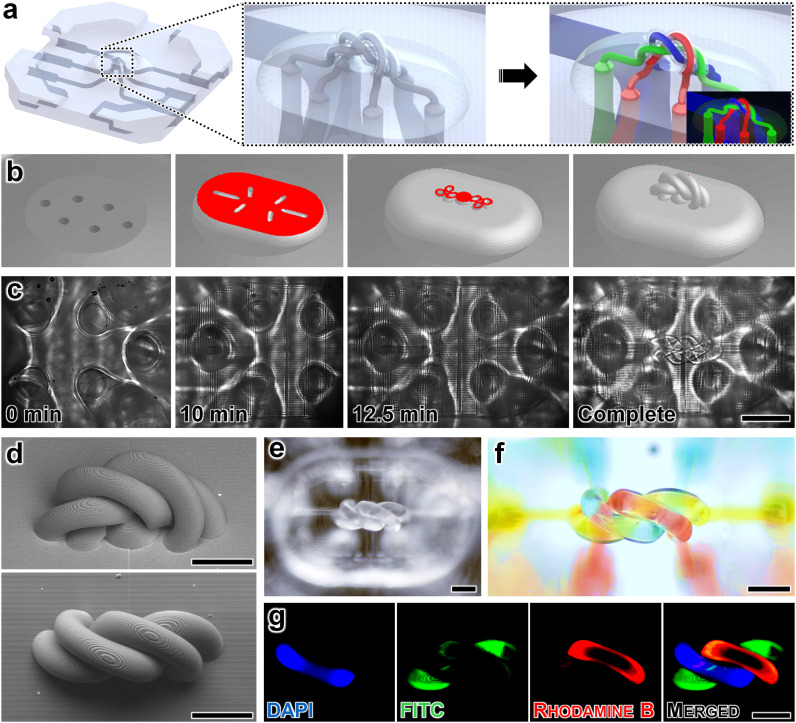
Fabrication results for *es*DLW 3D printing of three spiralled, intertwined microfluidic vessels using UpFlow photomaterial atop an LCD 3D-printed base microfluidic chip. (a) Conceptual illustration of the three intertwined microvessels atop the LCD 3D-printed microchip with expanded view before (left) and after (right) independent loading of distinct fluorescently labelled fluids. (b) CAM simulations. (c) Corresponding micrographs of the *es*DLW 3D printing process of the intertwined microvessel structure directly atop the LCD 3D-printed base microchip. Total print time <15 min; scale bar = 500 μm (see also ESI† Movie S4). (d) SEM micrographs of fabrication results. (Top) Tilted view. (Bottom) Top view. Scale bars = 250 μm. (e and f) Brightfield micrographs of the intertwined microvessel structures: (e) as printed, and (f) after microfluidic loading of coloured dyes. Scale bar = 250 μm. (g) Fluorescence micrographs of the intertwined microvessels independently loaded with distinct fluorescently labelled fluids. Blue = DAPI, green = FITC, red = rhodamine B; scale bar = 250 μm.

Fully intertwined microvessels are prevalent throughout tissue and organ systems *in vivo*, thus, we also explored the capacity for the presented strategy to enable such structures to be resolved *in vitro*. Specifically, we designed spiralled, intertwined microvessels comprising three discrete tortuous microfluidic vessel structures—each with an ID of 80 μm and a wall thickness of 10 μm—and investigated their manufacturability using an alternative (non-PDMS) photomaterial, UpFlow (UpNano), along with their ability to isolate distinct fluorescently labelled microfluidic flow streams (Fig. 3a). CAM simulations and corresponding micrographs captured during the *es*DLW process for 3D printing all three microvessels simultaneously—atop an LCD 3D-printed base microchip with six externally accessible top ports—are presented in Fig. 3b and c, respectively, which corresponded to a total print time of less than 15 minutes (ESI† Movie S4). SEM micrographs of *es*DLW-printed microvessels revealed effective fabrication (Fig. 3d). To evaluate the microfluidic integrity of the tortuous, fully intertwined microvessels based on their ability to maintain discretised flow, we configured the device such that: (i) one microvessel was connected to an input with a DAPI-dyed fluid; (ii) a different microvessel was connected to an input with a FITC-dyed fluid; and (iii) a third microvessel was connected to an input with a rhodamine B-dyed fluid (Fig. 3a). We used a similar experimental setup for imaging the intertwined microvessels with distinctly coloured dyes under brightfield microscopy (Fig. 3e and f). Fluorescence micrographs of the intertwined microvessels revealed uncompromised integrity of the microfluidic flow streams without any visible indications of undesired leakage or cross-contamination between discrete microvessels (Fig. 3g). Given the challenges in fabricating such fully intertwined microvessel-like structures at these length scales using alternative manufacturing approaches, these findings indicate that the presented *es*DLW strategy could offer unique utility for using a variety of materials to construct organ-on-a-chip systems requiring physiologically relevant 3D microfluidic vessels.

### Microfluidic burst-pressure studies

**Fig. 4:**
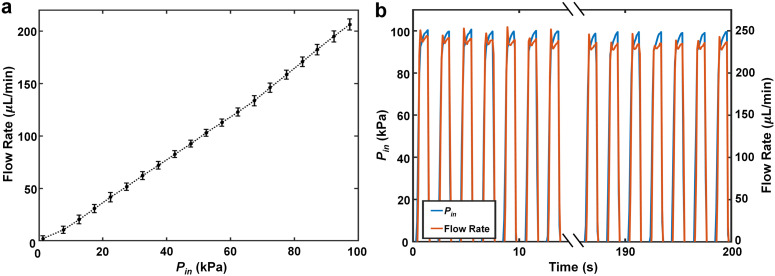
Quantified experimental results for microfluidic burst pressure investigations for 3D PDMS serpentine microvessels. (a) Linear microfluidic burst-pressure testing (*n* = 3 distinct devices). Error bars = S.D. (b) Representative cyclic burst-pressure experiments (*n* = 100 cycles) corresponding to input pressures (*P*_in_) targeting 100 kPa.

An important measure of manufacturing efficacy is the capacity for the system to withstand the forces associated with microfluidic loading without undesired detachment of the *es*DLW-printed microvessel structure from the LCD-printed base microfluidic device. To test this mechanofluidic integrity, we performed two sets of burst-pressure studies, corresponding to both linear and cyclic microfluidic burst-pressure testing. Both experimental observations and quantified results for linear burst-pressure testing did not reveal any indications of undesired leakage or burst events (*e.g.*, drastic increases in flow rate upon reaching a certain pressure) (Fig. 4a). Subsequently, we performed cyclic burst-pressure experiments in which *P*_in_ was set at 100 kPa for 5 seconds and then 0 kPa for 5 seconds repeatedly (*n* = 100 cycles). Akin to the linear burst-pressure tests, we observed microbeads flowing through microvessels and exiting either out the opposing end of the vessel or *via* the various arrayed micropores (but not the interface) during the cyclic burst-pressure experiments. Quantified results for the cyclic testing corroborated these findings, with the flow rates remaining consistent throughout the cyclic burst-pressure experiments (Fig. 4b). In combination, these results suggest uncompromised interface integrity between the *es*DLW-printed PDMS microvessels and the LCD-printed base microfluidic chip.

### Microfluidic extravasation studies

**Fig. 5:**
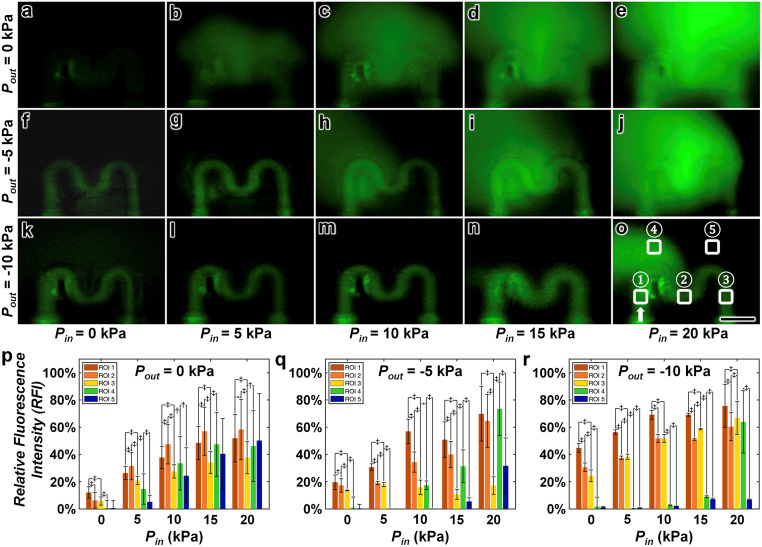
Experimental results for microfluidic extravasation studies with a 10% FITC solution. (a–o) Representative fluorescence micrographs of 3D PDMS microvessels with arrayed micropores (diameter = 5 μm) under varying *P*_in_ and output pressure (*P*_out_) conditions. *P*_out_ = (a–e) 0 kPa, (f–j) −5 kPa (vacuum), and (k–o) −10 kPa (vacuum); *P*_in_ = (a, f and k) 0 kPa, (b, g and l) 5 kPa, (c, h and m) 10 kPa, (d, i and n) 15 kPa, and (e, j and o) 20 kPa. (o) Arrow denotes input direction; numbered regions denote “regions of interest (ROIs)”; scale bar = 200 μm (see also ESI† Movie S1). (p–r) Quantified results for relative fluorescence intensity (RFI, eqn (1)) at each ROI corresponding to varying *P*_in_ and *P*_out_ conditions. *P*_out_ = (p) 0 kPa, (q) −5 kPa, and (r) −10 kPa. Negative values for *P*_out_ indicate vacuum pressure applied at the outlet; error bars = S.D.; *, †, and ‡ denote *p* < 0.05, 0.01, and 0.001 statistically significant differences, respectively.

The ability to design the 3D microvessels with arrayed micropores offers a potential pathway to extravasation-based research and applications; however, extravasation phenomena (*e.g.*, fluid flow out the micropores) is fundamentally related to both the magnitude of *P*_in_ as well as the output vacuum pressure (*P*_out_). To provide insight into this interplay, we performed two sets of microfluidic experiments in which both *P*_in_ and the magnitude of vacuum pressure applied at the outlet were increased. First, we infused a FITC solution into the microvessel while varying both *P*_in_ and *P*_out_. Experimental results for these microfluidic extravasation studies revealed three key trends (Fig. 5; ESI† Movie S1). First, for cases in which vacuum pressure was not applied at the outlet (*i.e.*, *P*_out_ = 0 kPa), we observed the highest rates of fluid flow out of the microvessel *via* the arrayed micro-pores, which appeared to increase directly with the magnitude of *P*_in_ (Fig. 5a–e). Second, by applying a 5 kPa vacuum pressure to the outlet (*i.e.*, *P*_out_ = −5 kPa), we found that fluid flow was maintained within the microvessel under the condition that the magnitude of *P*_in_ did not exceed that of *P*_out_ (*i.e.*, |*P*_in_| ≤ |*P*_out_|) (Fig. 5f and g). Lastly, for cases in which this condition was not met (*i.e.*, |*P*_in_| > |*P*_out_|), fluid flowed out of the microvessel *via* the arrayed micropores at rates that increased as the difference between *P*_in_ and *P*_out_ increased (Fig. 5h–j). Results from experiments in which we applied a 10 kPa vacuum pressure to the outlet (*i.e.*, *P*_out_ = −10 kPa) corroborated these two trends for the quasi-equilibrium pressurization (*i.e.*, |*P*_in_| = |*P*_out_|) representing the delineator in flow behaviour (Fig. 5k–o; ESI† Movie S1).

To quantify these microfluidic extravasation results, we measured the fluorescence intensities for each case corresponding to five ROIs: (1) adjacent to the entrance of the microvessel, (2) at the midpoint of the vessel, (3) preceding the exit of the microvessel, (4) above the first bend of the microvessel, and (5) above the second bend of the microvessel (Fig. 5o). The quantified results were consistent with the experimental observations, but also revealed two additional trends. First, the fluorescence intensity within the microvessel generally decreased as the fluid flowed from the vessel entrance (ROI_1_) to the exit (ROI_3_), likely due to dilution effects caused by micropore permeation along the flow path (Fig. 5p–r). Second, the initial onset of microfluidic extravasation (*e.g.*, |*P*_in_| − |*P*_out_| ≥ 5 kPa) led to higher fluorescence intensities above the first bend of the microvessel (ROI_4_) compared to that above the second bend of the microvessel (ROI_5_) (Fig. 5p–r). One basis for this behaviour is the relative fluidic resistance out the arrayed micropores along the proximal region of the microvessel (*e.g.*, from ROI_1_ to ROI_2_) compared to the distal region of the microvessel approaching the outlet (*e.g.*, from ROI_2_ to ROI_3_).

To provide an additional study with relevance to bacterial extravasation,^62^ we performed a second set of microfluidic extravasation experiments using a suspension of 1.0 μm microbeads (Thermo Fisher Scientific). Experimental results were consistent with those for the fluorescently labelled solution, but provided enhanced visualization of flow behaviour both through the microvessel as well as out of the microvessel *via* the arrayed micropores (ESI† Fig. S1; Movie S5). In combination, these findings indicate that both *P*_in_ and *P*_out_ can be modulated on-demand to control the microfluidic extravasation dynamics, thereby providing a means to regulate chemical and/or molecular exchanges between the interior and exterior of the microvessels and, in turn, simulate physiological conditions for studying cellular responses to varying microenvironmental cues.

### Cell studies

**Fig. 6:**
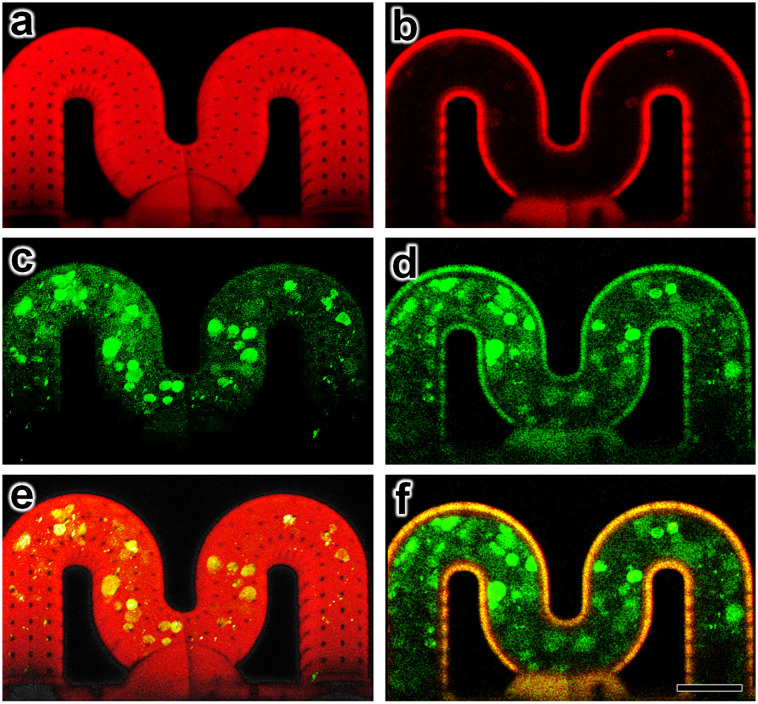
Experimental results for 24 hour culture of MDA-MB-231 cells inside the 3D PDMS microvessel. (a and b) Overlay of microvessel and propidium iodide (PI) (red), (c and d) GFP-expressing MDA-MB-231 epithelial breast cancer cells (green), and (e and f) merged images corresponding to (a, c and e) maximum intensity projection of z-stack series and (b, d and f) single slice at centre cross section. Scale bar = 100 μm (see also ESI† Movie S6).

Lastly, as a demonstrative example with relevance to OOC applications, we performed experiments in which mammalian cells were seeded in the 3D PDMS microvessels with arrayed micropores. The MDA-MB-231 epithelial breast cancer cell line is widely applied to study breast cancer metastasis due to its high metastatic abilities, therefore we apply it here to explore biological OOC applications. Experimental results for cell loading, adhesion, and viability using the MDA-MB-231 epithelial breast cancer cell line revealed that the 3D PDMS microvessels supported cell culture. Specifically, after 24 hours of culture on the inner lumen of the 3D PDMS microvessels, we observed that the MDA-MB-231 cells adhered to the vessel walls and remained viable, suggesting sufficiently low acute microvessel cytotoxicity and effective cell adhesion to the surface after coating (Fig. 6; ESI† Movie S6). The results also suggest that the micropores can be designed to ensure cell localization within the microvessel, and limit cell extravasation without stimulation, thereby providing a relatively stable microenvironment for cell growth. One observation was that the IP-PDMS exhibited a degree of auto-fluorescence (Fig. 6), which we did not observe for either the LCD-printed base microchip material (clear microfluidic resin) or the material used to print the intertwined microvessels (UpFlow) (Fig. 3g). Thus, auto-fluorescence may serve as a determinant in the selection of DLW-compatible photomaterial for microvessel construction.

## Conclusions

In this work, we introduced and demonstrated a novel hybrid strategy for additive manufacturing of PDMS-based microvessels featuring sophisticated 3D architectures and custom-designed micropores at biologically relevant scales. This fabrication approach, which combines VPP and *es*DLW, offers advantages in geometric versatility compared to traditional fabrication techniques, such as soft lithography and DIW-based approaches. The ability to produce fully 3D structures with precise control over geometrical parameters surpasses the limitations of planar architectures commonly associated with conventional methods. Moreover, the integration of *es*DLW with VPP allows for the seamless fabrication of complex microfluidic networks that can mimic the intricate vascular structures found *in vivo*. It is important to note that for the presented results based on the use of the Nanoscribe Photonic Professional GT2 DLW printer (acquired in 2014), such print speeds can be further accelerated—without compromising print resolution—using state-of-the-art DLW 3D printers. For example, using the UpNano NanoOne 1000 DLW 3D printer, we successfully DLW-printed the microvessels in under 30 minutes (ESI† Movie S7), and these microvessels similarly supported cell culture (ESI† Fig. S2; Movie S8). The ability to regulate microvessel permeation dynamics through input and output pressure modulation offers a powerful tool for investigating cellular behaviours under physiologically relevant conditions, such as for cancer cell extravasation and intravasation studies as well as additional cases that rely on microenvironmental control (*e.g.*, mimicking circulating tumour cell behaviours in venules during cancer metastasis). In combination, the strategy reported here could open the door to entirely new classes of OOC technologies for applications ranging from drug screening to biological science.

## Data availability

The data supporting this article have been included as part of the ESI.† Additional data are available upon request.

## Author contributions

All authors conceived the methodologies in the manuscript. X. X. led the efforts to design, fabricate, test, and analyze data for the microvessel. Y. Q. contributed to cell experiment. C. C. contributed to the LCD 3D-printed microfluidic chip design and fluidic testing. M. C. contributed to test and data quantification. P. C. contributed to the intertwined microfluidic structure design. A. M. C. contributed to the chip design modifications. All authors prepared and reviewed the manuscript.

## Conflicts of interest

There are no conflicts to declare.

## Supplementary Material

LC-025-D4LC01051E-s001

LC-025-D4LC01051E-s002

LC-025-D4LC01051E-s003

LC-025-D4LC01051E-s004

LC-025-D4LC01051E-s005

LC-025-D4LC01051E-s006

LC-025-D4LC01051E-s007

LC-025-D4LC01051E-s008

LC-025-D4LC01051E-s009
