# Supplementary material for: 3D nanoprinting of PDMS microvessels with tailored tortuosity and microporosity via direct laser writing
Source: Lab Chip. 2025 Mar 12;25(8):1947–58. doi: 10.1039/d4lc01051e (PMC11921864; doi:10.1039/d4lc01051e)
Supplement: LC-025-D4LC01051E-s009 [file LC-025-D4LC01051E-s009.pdf]

Supplementary Information (SI) for Lab on a Chip.
This journal is © The Royal Society of Chemistry 2025

SUPPORTING INFORMATION

3D Nanoprinting of PDMS Microvessels with Tailored Tortuosity and Microporosity *via* Direct Laser Writing

Xin Xu[a], Yunxiu Qiu[b], Chen-Yu Chen[c,d], Molly Carton[a], Paige M.R. Campbell[c], A. Muhaymin Chowdhury[a], Bidhan C. Bandyopadhyay[e], William E. Bentley[c,d], Bryan Ronain Smith[b,f] and Ryan D. Sochol [a,c,d,g,h,]∗

[a.] Department of Mechanical Engineering, University of Maryland, College Park, MD, 20742, USA.

[b.] Institute for Quantitative Health Science and Engineering, Department of Chemical Engineering and Material Science, Michigan State University, East Lan-sing, MI, 48824, USA.

[c.] Fischell Department of Bioengineering, University of Maryland, College Park, MD, 20742, USA.

[d.] Robert E. Fischell Institute for Biomedical Devices, University of Maryland, College Park, MD, 20742, USA.

[e.] Veterans Affairs Medical Center, Washington, D.C., 20422, USA.

[f.] Department of Biomedical Engineering, Michigan State University, East Lansing, MI, 48824, USA.

[g.] Maryland Robotics Center, University of Maryland, College Park, MD, 20742, USA.

[h.] Institute for Systems Research, University of Maryland, College Park, MD, 20742, USA.

* Ryan D. Sochol, 2147 Glenn L. Martin Hall, University of Maryland, College Park, MD 20742, USA; rsochol@umd.edu

Supporting Materials:

Figures S1–S2

Tables S1–S7

Movies S1–S8

SUPPORTING FIGURES

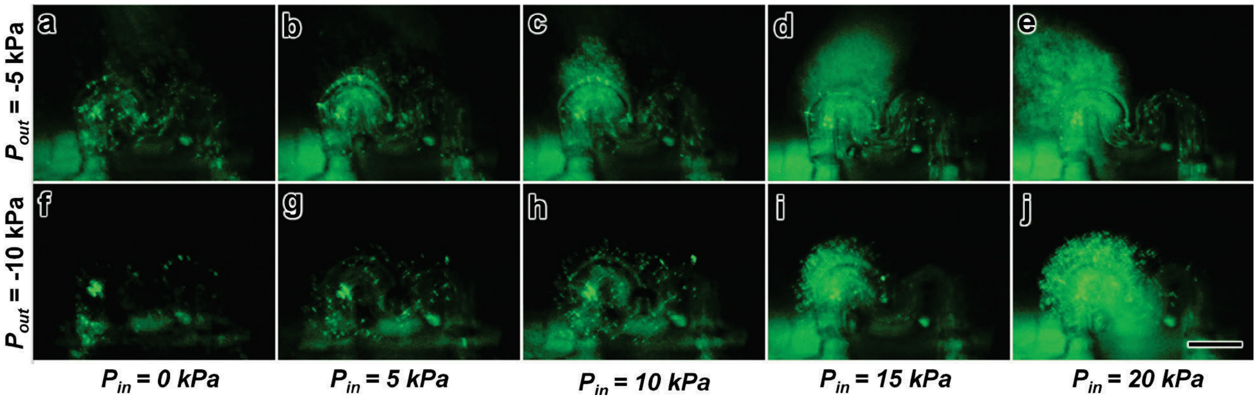

Figure S1 | Experimental results for microfluidic extravasation studies with a suspension of 1.0-μm-diameter microbeads. **(a–j)** Representative fluorescence micrographs of 3D PDMS microvessels with arrayed micropores (diameter = 5 μm) under varying input pressure (P_{in}) and output pressure (P_{out}) conditions. P_{out} = **(a–e)** -5 kPa (vacuum), and **(f–j)** -10 kPa (vacuum); P_{in} = **(a,f)** 0 kPa, **(b,g)** 5 kPa, **(c,h)** 10 kPa, **(d,i)** 15 kPa, and **(e,j)** 20 kPa. Scale bar = 200 μm (see also **ESI† Movie S5**).

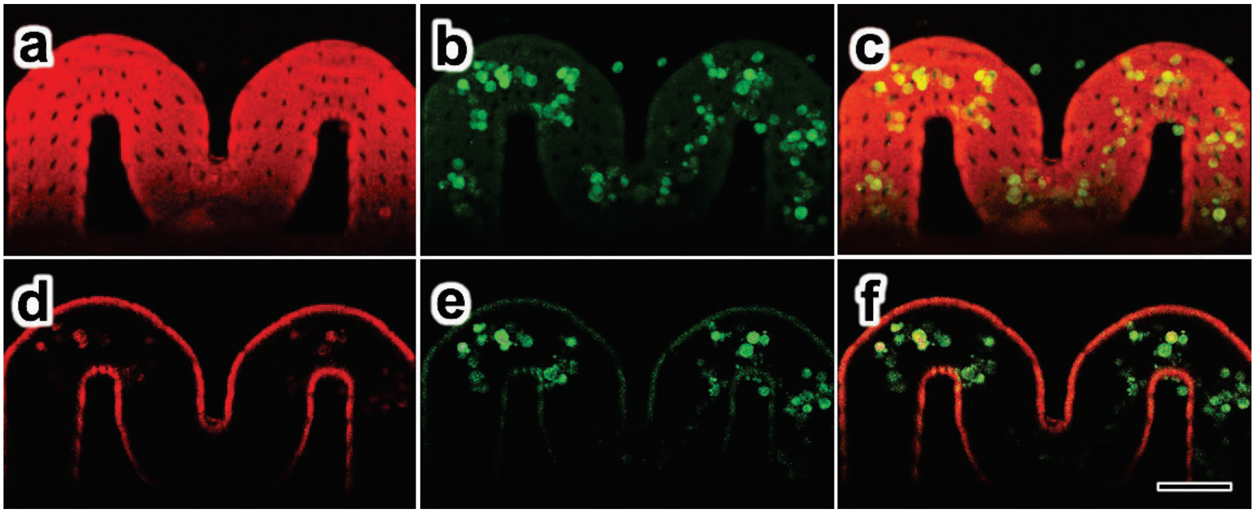

Figure S2 | Experimental results for 24-hour culture of MDA-MB-231 cells inside a 3D PDMS microvessel printed using the UpNano NanoOne 1000 DLW 3D printer. **(a–c)** Maximum intensity projection of z-stack series and **(d–f)** single slice at center cross section. **(a,d)** Microvessel (*red*). **(b,e)** GFP-expressing MDA-MB-231 epithelial breast cancer cells (*green*). **(c,f)** Merged images. Scale bar = 100 μm (see also **ESI† Movie S8**).

Table S1. Parameter Settings for Elegoo Mars 3 LCD 3D printer Slicing Tool to 3D Print the Base Microfluidic Device.

**Parameter**	**Value**
Layer Thickness	30 μm
Layer Exposure time	1.9 s
Base Layer Count	3
Base Layer Exposure Time	15 s
Buffer Layer Count	5

Table S2. Parameter Settings for Describe Software (Nanoscribe) to 3D Print the PDMS-Based Serpentine Microvessel Structure with Nanoscribe Photonic Professional GT2 DLW printer (10× Objective).

**Parameter**	**Value**
Slicing Distance	0.3 μm
Hatching Distance	300 nm
Hatching Angle	0°
Hatching Angle Offset	90°
Power Scaling	1.0
Solid Laser Power	50 mW
Solid Scan Speed	100,000 μm/s
Hatch Lines Direction	Alternative
Scan Mode	Galvo
Z Axis Movement	Z Drive

Table S3. Parameter Settings for Think3D Software (UpNano) to 3D Print the Base of the Intertwining Microvessel Structure with NanoOne 1000 DLW Printer (10× Objective).

**Parameter**	**Value**
Slice Mode	Voxel
Infill Mode	Adaptive
Layer Height	5 μm
Fine Infill Power	220 mW
Fine Line Distance	0.5 μm
Fine Infill Speed	600 mm/s
Coarse Infill Power	400 mW
Coarse Line Distance	4 μm
Coarse Infill speed	600 mm/s

Table S4. Parameter Settings for Think3D Software (UpNano) to 3D Print the Intertwining Microvessel Structure with NanoOne 1000 DLW Printer (10× Objective).

**Parameter**	**Value**
Slice Mode	Voxel
Infill Mode	Fine
Layer Height	5 μm
Fine Infill Power	220 mW
Fine Line Distance	0.5 μm
Fine Infill Speed	600 mm/s

Table S5. Parameter Settings for Think3D software (UpNano) to 3D Print the Base of the Serpentine Microvessel Structure with NanoOne 1000 DLW Printer (20× Objective).

**Parameter**	**Value**
Slice Mode	Simple
Infill Mode	Coarse
Layer Height	1 μm
Fine Infill Power	85 mW
Fine Line Distance	1.6 μm
Fine Infill Speed	55 mm/s

Table S6. Parameter Settings for Think3D software (UpNano) to 3D Print Serpentine Microvessel Structure with NanoOne 1000 DLW Printer (20× Objective).

**Parameter**	**Value**
Slice Mode	Voxel
Infill Mode	Fine
Layer Height	1 μm
Fine Infill Power	90 mW
Fine Line Distance	0.6 μm
Fine Infill Speed	55 mm/s

Table S7. Properties of Objective Lenses

Property	Nanoscribe 10×	UpNano 10×	UpNano 20×
Numerical Aperture	0.3	0.4	0.7
Working Distance	700 μm	3100 μm	350 μm
Field of View (Diameter)	1000 μm	2000 μm	1000 μm
Immersion Media	air	air	water
Theoretical Lateral (a_{xy}) Resolution	≤ 1.6 μm	≤ 730 nm	≤ 420 nm
Theoretical Axial (a_z) Resolution	≤ 25.4 μm	≤ 9.2 μm	≤ 2.9 μm

SUPPORTING MOVIE CAPTIONS

Movie S1 | Experimental results for microfluidic extravasation studies with a 10% fluorescein-5-isothiocyanate (FITC) solution for 3D PDMS microvessels with arrayed micropores (diameter = 5 μm) under varying input pressure (P_{in}) and output pressure (P_{out}) conditions.

Movie S2 | Removal of the build substrate following LCD 3D printing of 12 base microfluidic chips simultaneously. Print time ≈ 30 min.

Movie S3 | The "*ex situ* DLW (*es*DLW)" 3D printing process for fabricating a PDMS microvessel directly atop two of the externally accessible top fluidic ports of the base microfluidic chip using the Nanoscribe Photonic Professional GT2 DLW printer. **(*Left*)** Computer-aided manufacturing (CAM) simulations. **(*Right*)** Corresponding micrographs of the *es*DLW 3D printing process. Total print time ≈ 38 min.

Movie S4 | The "*ex situ* DLW (*es*DLW)" 3D printing process for fabricating fully intertwining microvessels directly atop six of the externally accessible top fluidic ports of the base microfluidic chip using the UpNano NanoOne 1000 DLW printer. Total print time ≈ 15 min.

Movie S5 | Experimental results for microfluidic extravasation studies with a suspension of 1.0 μm Fluoro-Max dyed green aqueous fluorescent particles for 3D PDMS microvessels with arrayed micropores (diameter = 5 μm) under varying P_{in} and P_{out} conditions.

Movie S6 | 3D view of 24-hour culture results for GFP-expressing MDA-MB-231 epithelial breast cancer cells (*green*) seeded within a 3D PDMS microvessel (*red*) printed using the Nanoscribe Photonic Professional GT2 DLW printer.

Movie S7 | The *es*DLW 3D printing process for fabricating a PDMS microvessel directly atop two of the externally accessible top fluidic ports of the base microfluidic chip using the UpNano NanoOne 1000 DLW 3D printer. Total print time ≈ 14 min.

Movie S8 | 3D view of 24-hour culture results for GFP-expressing MDA-MB-231 epithelial breast cancer cells (*green*) seeded within a 3D PDMS microvessel (*red*) printed using the UpNano NanoOne 1000 DLW 3D printer.
